# Animal transport as regulated in Europe: a work in progress as viewed by an NGO

**DOI:** 10.1093/af/vfac010

**Published:** 2022-03-17

**Authors:** Nikita Bachelard

**Affiliations:** La Fondation Droit Animal, Ethique et Sciences (LFDA), Paris, France

**Keywords:** animal transport, animal welfare, enforcement, European Union, livestock, regulation

**Implications**

- Regulation 1/2005 on the protection of animals during transport entered into force 15 years ago.
- Scientific literature, official reports from the European Commission, and nonofficial reports from NGOs underline that the regulation is not being properly enforced, and thus does not always protect animals during transport, especially concerning fitness for transport, loading, and unloading.
- We argue that: 1- Inspections should be more frequent and improved to avoid transporting unfit animals; 2- The Regulation should protect all livestock species, including aquatic animals, with provisions for each species and stage of life; 3- The maximum duration for a journey should be set; and 4- Livestock exports should be phased out.

## Introduction

In early 2021, the media shed light on a confusing situation related to the transport of live cattle by sea. After departure from Spain on December 18, 2020, the two livestock vessels Karim Allah and Elbeik were refused entry into the Middle East ports and all other Mediterranean ports because of an alleged risk of blue-tongue disease. Then 895 bovines remained on the Karim Allah and 1,871 on the Elbeik in Spanish waters. The Karim Allah was finally allowed into the port of Cartagena at the end of February 2021. After having spent 79 days onboard, 22 bovines were dead on arrival. A week later, the remaining animals were declared unfit for transport by Spain’s official veterinaries and were euthanized. The Elbeik entered Cartagena after 3 months at sea with 189 dead animals onboard (Boada-Saña et al., 2021). Before euthanizing the remaining animals, inspectors described the animals as in poor health (dehydration, weight loss, lethargy), as reported by the law firm Joaquin Ortega Abogados (2021). Animal welfare nongovernmental organizations (NGOs) became very vocal about the situation that resulted in the suffering and death of many animals and claimed it could and should have been avoided.

These events occurred even though the European Union (EU) set up a number of requirements to protect animals during transport. The provisions are defined in Council Regulation (EC) No 1/2005 of 22 December 2004 on the protection of animals during transport and related operations and amending Directives 64/432/EEC and 93/119/EC and Regulation (EC) No 1255/97 (hereafter named Regulation 1/2005).

Based on Eurostat’s reference database, the NGO Eurogroup for Animals (2021) estimated that in 2019, over 1.6 billion livestock (mainly ovine, bovines, poultry, and pigs) were transported alive across the EU and beyond its borders by road, sea, rail, and air for trade purposes. Fish exports amounted to 57,523 tons (not expressed in heads), with 93% within the EU.

Animals are transported alive mainly for slaughter, fattening, and breeding (DG SANTE, 2020a). Economic considerations (free trade and competition, specialization of production systems), or cultural considerations (consumer preferences, religious considerations) drive livestock transport over long distances (Massot et al., 2021). Often, regional production differs from regional consumption (Baltussen et al., 2017). Another driving cause of extended duration spotlighted by Eurogroup for Animals (2019) is the decrease in the number of slaughterhouses in Europe in the last decades leading to longer distances to reach facilities.

Live transport may pose several threats to animal welfare, during loading and unloading and during transport per se in relation to grouping, density, and handling (Appleby et al., 2008). In addition, the welfare of animals decreases with journey duration (Schwartzkopf-Genswein et al., 2012; Padalino, 2015; Padalino et al., 2018).

For decades, animal welfare NGOs have exposed footages and reports of poor welfare of animals transported alive over long journey (see for example Animal Welfare Foundation, 2017). In the meantime, animal welfare has become a concern for EU citizens with, according to the last Eurobarometer on animal welfare (DG SANTE, 2016) , 94% of Europeans thinking that it is important to protect the welfare of farmed animals (DG SANTE, 2016).

We propose an overview of Regulation 1/2005 as regards its contribution to improving animal protection, as well as its limits. We start by explaining the regulation and its main objectives. We then explore the claims about its adequacy to ensure animal protection or lack thereof, and investigate propositions to improve the situation overall. We focus on road and sea transport. This article reflects the opinion of an NGO based on scientific evidence and on official as well as nonofficial reports on animal transport in Europe.

## European Regulation on Live Transport: Main Goals

### Legislative and political context

A first piece of legislation on the protection of animals during transport (Directive 91/628/EEC) was set in 1991 to harmonize the protection of animals transported in all EU Member States (MS). Several pieces of legislation followed: Directive 95/29/EC, Council Regulation (EC) No 1255/99, and Council Regulation (EC) No 411/98. In the early 2000’s, reports by the Food and Veterinary Office (FVO)—a division of the European Commission, now called Health and Food Audits and Analysis—as well as investigations from animal welfare NGOs revealed poor improvements of the welfare of animals during transport despite legislative requirements had been set (Corson and Anderson, 2008). Council Regulation (EC) No 1/2005 amended the former directive and regulations in order to improve enforcement (Cussen, 2008). The Regulation is transposed word for word in MS legislation as opposed to a directive.

### Scope and objectives of Regulation 1/2005

According to article 1, Regulation 1/2005 applies to “*the transport of live vertebrate animals carried out within the Community*”. The aim of the regulation is to prevent “*injury or undue suffering*” to animals during transport (Article 3), to limit the transport of animals during long journey as far as possible (Recital 5), and “*to safeguard the welfare and health of animals during and after transport*” (Recital 6).

Transport from and to veterinary premises does not fall under the scope of the Regulation. The Regulation applies to all stakeholders involved in the transport of live animals, within, entering, or leaving the EU (Chapter II). MS are allowed to take stricter national measures to further improve the welfare of animals during transport (Article 1).

### Main requirements of Regulation 1/2005

Regulation 1/2005 requires animals to be fit for transport, suitable means for transport and for the loading and unloading, trained staff for handling animals, adequate flow of transport, and care given to animals (space, food, and water).

Regulation 1/2005 sets provisions by animal species (except for fish), by type of transport (road, sea, air), by type of actors (transporters, keepers, operators, assembly centers, official national competent authorities...). The Regulation distinguishes between a journey lasting 8 h or less and a “long journey” that exceeds 8 h. Long journeys have to comply with more provisions (related to vehicle certifications, inspections, authorizations, animal welfare…).

The journey starts when the first animal is loaded into the vehicle and ends when the last animal is unloaded from the vehicle at arrival. In 2015 and 2016, the Court of Justice of the European Union (CJEU) ruled that companies transporting live animals from the EU to a third country must respect the provisions of Regulation 1/2005 for all parts of the journey, including the parts taking place outside Europe (CJEU cases C-424/13 and C‑383/16).

## Efforts to Implement Regulation 1/2005 and Impact on Animal Welfare

### European Commission’s audits and Animal Welfare Platform

In the period 2009–2015, FVO carried out 40 audits in MS regarding Regulation 1/2005. FVO also performed follow-ups to check how MS dealt with the recommendations. In addition, they audited the Turkey-Bulgarian border in 2017–2018 as well as key MS concerned by the transport to third countries (Baltussen and Wagenberg 2018). DG SANTE produced overview reports, including on fitness for transport, on exportation by road, and on exportation by sea. In its first overview report, DG SANTE (2015) pointed that animals unfit for transport are still regularly transported; this may be due to poor communication between official departments and authorities as well as to sanctions being not dissuasive enough. In its second overview report, DG SANTE (2020a) concluded in a high level of compliance with the rules for transports inside the EU but not for transports towards third countries. More specifically, recurrent issues are noticed at the Turkish-Bulgarian border and during the part of the journey outside the EU. Main causes are high temperatures and incompliance with the EU Regulation in third countries (DG SANTE, 2020a). The third report on sea transport concluded on staff not suitably qualified and on transport approved despite irregularities. Loading of animals works well in most cases but when it doesn’t, there is no contingency plan and no available animal facilities. Finally, the welfare of animals onboard is unknown (DG SANTE, 2020b).

The European Commission also set up an Animal Welfare Platform to gather governments, scientists, businesses, and NGOs together to exchange good practices in animal welfare (https://ec.europa.eu/food/animals/animal-welfare/eu-platform-animal-welfare_en). A network of national contact points and the Animal Welfare Platform increased harmonization among MS. Overall, Regulation 1/2005 have reached the objective of a better harmonization among MS in the sector (Baltussen and Wagenberg 2018).

### Training and guides

The regulation imposes training for staff handling the animals. Since 2005, the European Commission has set up a training program called Better Training for Safer Food (Agra CEAS Consulting Ltd, 2018) to help compliance with EU legislation.

Practical Guidelines to Assess Fitness for Transport for adult bovines, pigs and Equidae have been developed to help reach compliance with the Regulation (Eurogroup for Animals et al., 2012, 2015 and World Horse Welfare et al., 2015).

As part of a European Commission’s Consortium of the Animal Transport Guides Project, guides to good practices for the transport of sheep, cattle, pigs, horses, and poultry have also been developed to help reach compliance with the Regulation (Consortium of the Animal Transport Guides Project, 2017). Therefore, efforts have been made by the EU and national competent authorities as well as various stakeholders to implement the Regulation.

### Impact in terms of animal welfare

According to the European Commission’s website, “*the number of animals transported with injury, or exhaustion significantly decreased*” (https://ec.europa.eu/food/animals/animal-welfare/main-achievements_en). However, no animal-based measures or good indicator on animal welfare exist to support this claim (Baltussen et al., 2011; Baltussen and Wagenberg, 2018).

Mortality and fitness for transport are used as indicators of welfare for transported animals. Mortality is only reported in scientific literature and no general conclusion can be drawn as regards improvements of animal welfare thanks to the implementation of Regulation 1/2005. Analysis of annual inspection reports from MS for 2014 and 2015 shows that fitness has the highest level of noncompliance (Baltussen and Wagenberg, 2018). To our knowledge, no analysis has been done for annual inspection reports from 2016 onward. The analysis of the inspection report from France reveals that in 2018, 41 penalties over 55 related to transport were for transporting unfit animals (Annex to the annual report on inspections carried out in France in 2018). Dahl-Pedersen et al. (2018) argued that the training of farmers, drivers, and veterinarians on recognizing fitness for transport of dairy cows may not be sufficient. There is thus still a need to improve the training of people transporting animals as well as the training of farmers and veterinarians who decide or advise to send animals.

The reduction of journey duration has a potential to improve welfare. Nevertheless, between 2009 and 2015, the number of long and very long journeys almost doubled from 72,000 to 125,000, and increased relatively more than the number of short journeys (Baltussen and Wagenberg, 2018). Between 2014 and 2017, based on Eurostat and TRACES (TRAde Control and Expert System) data, Eurogroup for animals (2019) reports an increase in trade flows of live animals both intra-EU and extra-EU.

In recent years, more and more MS adopted partial bans on the export of animals to Turkey or North Africa during summertime or at least summer heat waves (Eyes on Animals, 2019). During long journeys, risks of poor welfare due to heat stress are particularly high (Caulfield et al., 2014) so such decisions are positive steps for animal welfare.

Overall, data on animal transport is limited (Padalino et al., 2018) and not reliable enough (European Court of Auditors, 2018). Therefore, even though Regulation 1/2005 probably led to some progress in the welfare of animals during transport (Baltussen et al., 2011), the impact of the efforts to better implement Regulation 1/2005 on the welfare of animals remains unclear.

## Limits of EU Legislation to Protect Animals During Transport

Baltussen and Wagenberg (2018) noted that “*it is impossible to know if minimum requirements regarding animal welfare have been reached*” and reminded that “*NGOs still find examples of bad transports*”. In a recent joint statement, the Netherlands, Germany, and Luxembourg governments stated that “*Despite many efforts to improve compliance with the provisions of Council Regulation (EC) No 1/2005, and despite the fact that best practices exist, we conclude that the welfare of animals cannot be sufficiently guaranteed during these type of long journeys*” (Council of the European Union, 2021). The European Commission (2020) “*will revise the animal welfare legislation, including on animal transport […], to align it with the latest scientific evidence […], make it easier to enforce and ultimately ensure a higher level of animal welfare*”. In a press release from 3 December 2021, the European farmers’ organization Copa and Cogeca (2021) stated: “*There is a need for a revision after 16 years since it was first approved and a science-based update may further guarantee a harmonized enforcement and implementation of the Regulation 1/2005 across the Member States*”.

### Issues regarding fitness for transport

Regulation 1/2005 states that “*No animal shall be transported unless it is fit for the intended journey, and all animals shall be transported in conditions guaranteed not to cause them injury or unnecessary suffering*” (Annex I, Chapter 1, 1). However, there is no definition of “fitness for transport” (Herskin et al., 2020). The Regulation states that “*Animals that are injured or that present physiological weaknesses or pathological processes shall not be considered fit for transport*” (Annex I, Chapter 1, 2). This ability to assess fitness for transport is particularly crucial for culled animals (e.g. dairy cows) who usually are more vulnerable (Dahl-Pedersen et al., 2018). The European Commission observed that “*injured animals arrive on a daily basis to slaughterhouses in the European Union*” (DG SANTE, 2015). Inspections reports from 2014 and 2015 showed that “*fitness for transport is responsible for the largest percentage of infringements (28% and 43% respectively)*”.

### Issues at loading and unloading

**Figure 1.**
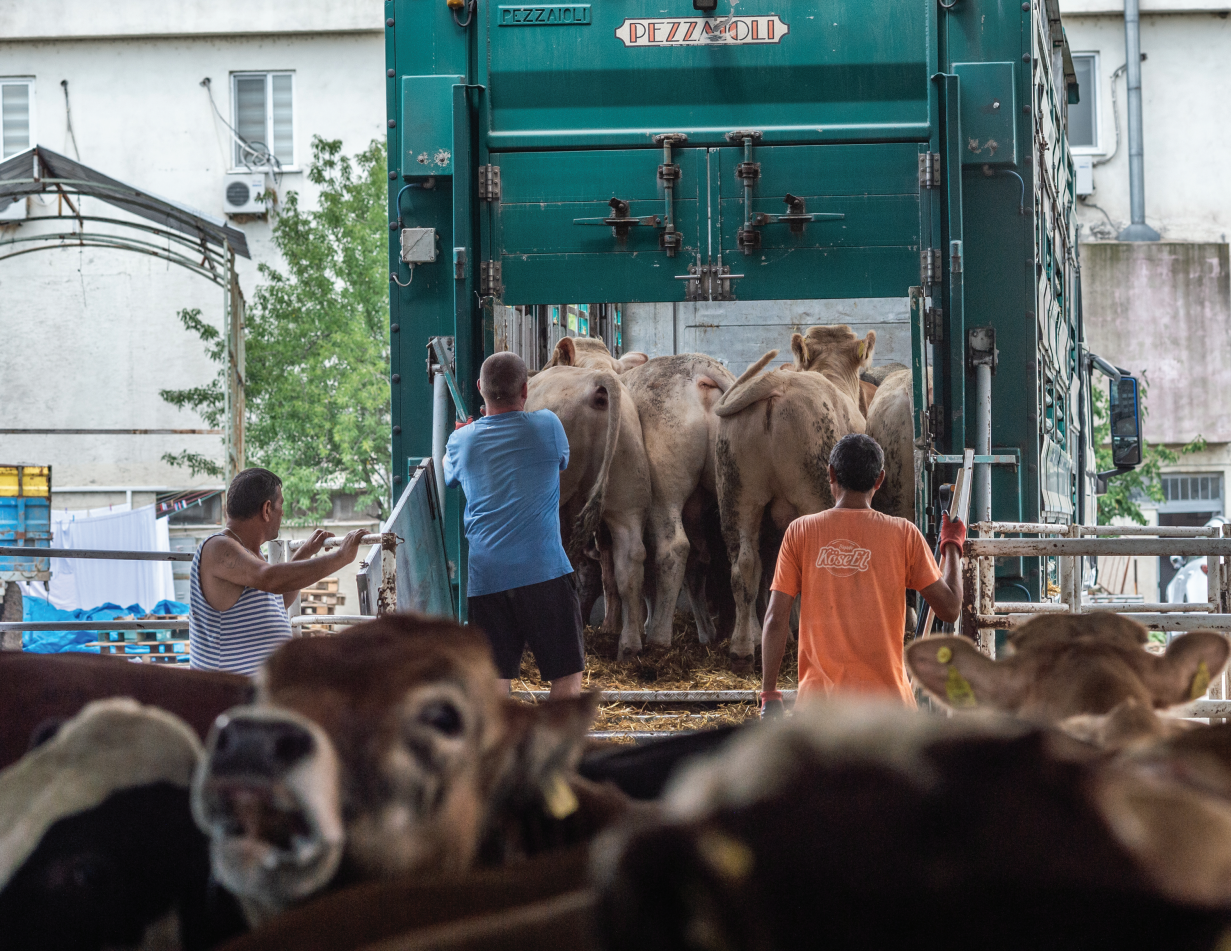
Cattle loaded back onto trucks after stopping at a feed station at the Bulgarian-Turkish border. 2018. Jo-Anne McArthur/Eyes on Animals/We Animals Media.

Loading and unloading can be particularly stressful for the animals because they are moved from a familiar environment to a new one and they are often mixed with unfamiliar individuals (van Staaveren et al., 2015; Broom, 2019; Hubbard et al., 2021; Figure 1). Other factors that can induce stress include handling, quality and safety of the facilities, and group size (Šímová et al., 2016). NGOs reported abuses towards animals at loading such as rough handling, e.g. using electric prod although it is forbidden (Animal Welfare Foundation, 2017). The prevalence of the problem remains unknown.

### Issues during transport

High stocking densities pose a risk to animal welfare during transport (González et al., 2012;Schwartkopf-Genswein et al., 2012). Overcrowding results in stress, falls, bruising and injuries, and inhibition of social and exploratory behaviors (Tarrant et al., 1988). Overcrowding is a frequent cause of infringement (Padalino et al., 2020). In addition to space allowance, inadequate partitions and insufficient headroom may lead to discomfort, improper ventilation, and injuries (Broom, 2008). Additionally, specifications of Regulation 1/2005 (Annex I) may not be adapted to specific types of animals. For instance, the space allowed by the Regulation to unweaned lambs is not enough to protect their welfare (Menchetti et al., 2021).

**Figure 2.**
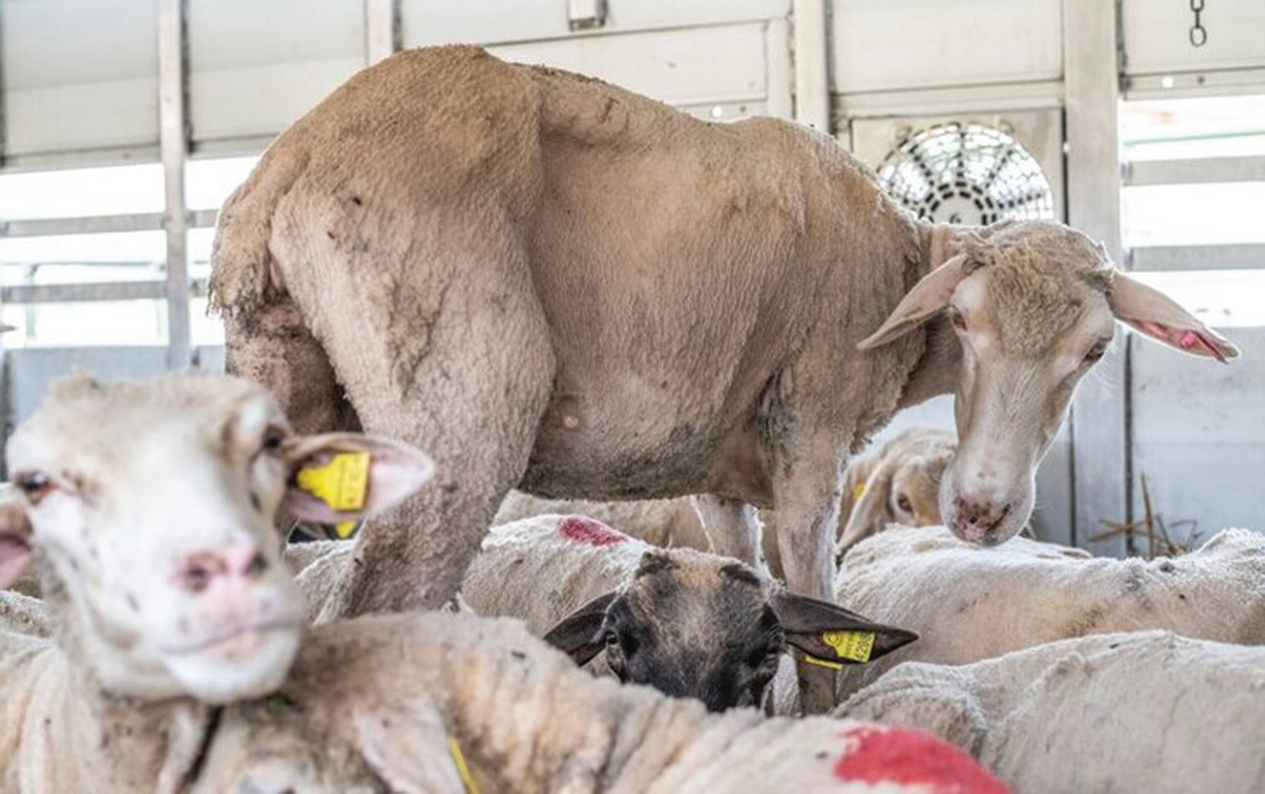
Sheep inside a transport truck parked at the Bulgarian-Turkish border. 2018. Jo-Anne McArthur/Eyes on Animals/We Animals Media.

Regulation 1/2005 requires the maintenance of “*a range of temperatures from 5°C to 30°C within the means of transport, for all animals, with a +/– 5°C tolerance*”. In summer time, temperatures often exceed 30°C in Southern Europe, Eastern Europe, the Middle East, and North Africa. At the Bulgarian-Turkish border, the second busiest of the world, long queues of vehicles wait to cross the border while temperatures exceed 30°C. Animals can be held there for days (DG SANTE, 2020a) (Figure 2). During long transports, animal welfare is likely to be very poor because of heat stress due to high temperatures combined with overcrowding, leading to distress and sometimes death (Caulfield et al., 2014).

Regulation 1/2005 defines maximum journey durations before a 24-h break is needed: 18 h with a 1-h break in the middle for unweaned animals, 24 h for pigs and equids, 28 h with a 1-h break in the middle for cattle, sheep, and goats, and poultry and rabbits may be transported up to 12 h without provision of feed and water. The journey can resume after the break. This can last as long as the transport requires and does not apply to sea transport. In a recent study analyzing TRACES data, Paladino et al. (2021) found that 20.9% of the analyzed and declared stops did not comply with the Regulation.

### Lack of data

As mentioned earlier, data on animal transport is limited. The European Court of Auditors (2018) reported that data on animal transport is very difficult to obtain because it is available only at a very local level. For long journeys involving more than one MS, consignment must be reported in TRACES but the database has limits (DG SANTE, 2020a). TRACES does not enable to identify if long journeys have been “limited as far as possible” as required (Dahl-Pedersen et al., 2021). The data cannot be broken down into relevant animal categories (unweaned calves, cull sow, etc.) (Dahl-Pedersen et al., 2021). TRACES does not provide data on animals transported within countries or directly outside the EU without passing through other MS (Dahl-Pedersen et al., 2021). Almost half of TRACES entries are irregular or not completely filled in (Padalino et al., 2021). In addition, TRACES database is not transparent and open access (Padalino et al., 2021).

## Difficulties in Enforcing Regulation 1/2005

The Council of the European Union (2019) expressed that “*rules need to be better enforced”*. The European Parliament (2019) “*[regretted] the fact that the degree of progress in implementation of Regulation (EC) No 1/2005 by Member States has been insufficient to meet the Regulation’s main objective […]*”.

Analyzing the sea transport events mentioned earlier, the accountability report from Joaquin Ortega Abogados (2021) concluded: “*Administrative errors, technicalities in the reporting of the origin of the bovines exported, and lack of agreement in the interpretation of the concepts of zone, region or country, generated this animal welfare crisis. […] Serious breaches, and the lack of coordination between the countries involved in the two crises, prevented the animals from being unloaded on the mainland and provided with the necessary rest and care*”.

### Checks and documentation

Regulation 1/2005 provides many requirements as well as mandatory inspections for compliance. The number of checks at departure, during transport, and at final destination decreased between 2009 and 2015 (Baltussen and Wagenberg, 2018). One reason for this decrease may be that some MS favored the risk-based approach for inspections, that is they check transport that they consider at risk according to predefined parameters, such as noncompliances during previous inspections, especially related to animal welfare, or transport that have not been inspected yet.

Most remaining noncompliances relate to animal fitness for transport or to documentation (Baltussen and Wagenberg, 2018). Infringements related to documentation tend to decrease over years whereas animal welfare infringements remain stable (Padalino et al., 2020).

The European Commission considers that “*many competent authorities approve the transport with incomplete or incorrect documentation*” (DG SANTE, 2020b). Padalino et al. (2021) found irregularities in almost half of the analyzed TRACES entries approved for transporting cattle. There is thus a need to improve checks before transport.

### Varying sanctions between MS

Each MS has its own legal system, especially concerning sanctions and this can lead to variations in enforcement (Baltussen and Wagenberg, 2018). For instance, transporting unfit animals can be charged with a penalty of €38,778 in Romania, €2000 in Italy, and €600 or a warning in Spain (Meriggi, 2020). These variations contribute “*to the difficulty for official veterinarians in managing infringements for interstate transport*” (Paladino et al., 2021).

### Animal welfare requirements

Transporters must provide authorities with a contingency plan in case of accident, brutal change of weather, sick or injured animals, mechanical breakdowns... However, “*few competent authorities, road transporters, and transport organizers have contingency plans for these situations*”, which can result “*in animals enduring long times in the vehicles, with negative consequences for their welfare*” (DG SANTE, 2020b).

Regarding sea transport, “*when [preloading inspection reports] reflected deficiencies, the vessel was still allowed to transport animals, although deficiencies were not always corrected before departure*” (DG SANTE, 2020b). As revealed by the NGO Animal Welfare Foundation (2017), most livestock vessels are former car ferries and cargo ship and their average age is 35 years while the lifetime of a cruise ship is 20 years. The vessels transporting animals have thus more chance than other vessels to encounter failures.

Persisting problem of inappropriate equipment of trucks is also often noted especially for the transport of unweaned calves (Baltussen and Wagenberg, 2018).

### Difficulties in applying EU rules for long transports

**Figure 3.**
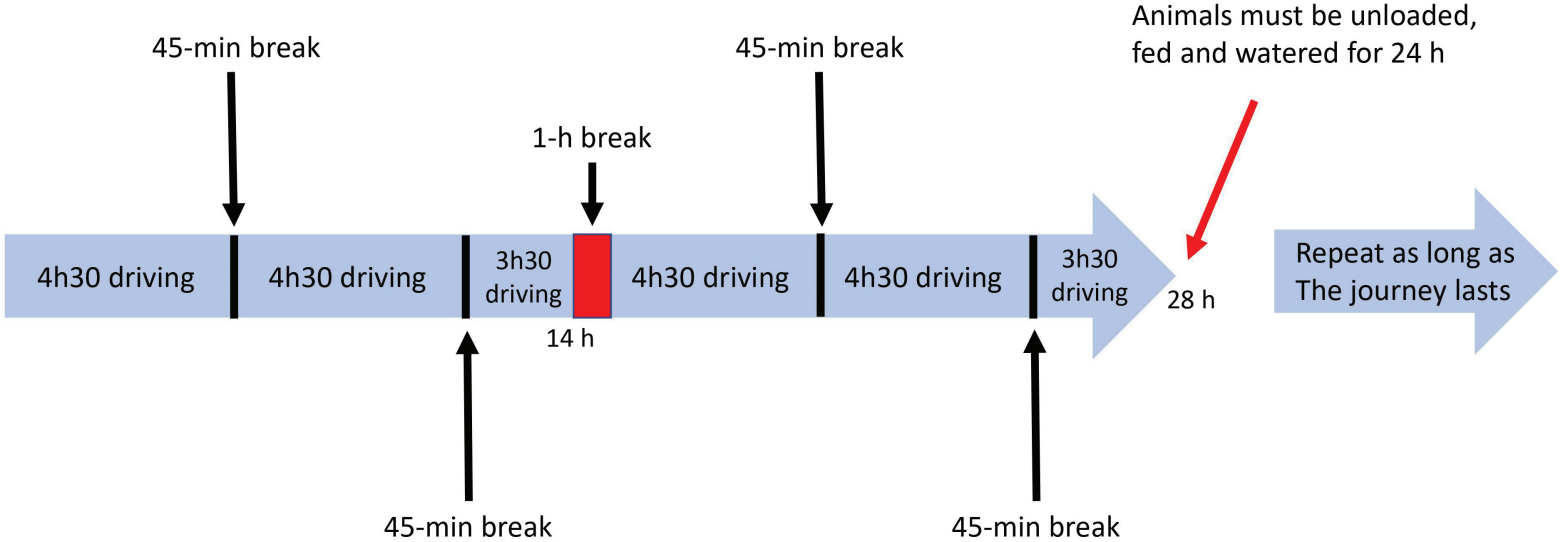
Example of the organization of a driver during the journey (according to CJEU C-469/14 and Duthoit (2017)).

Drivers have to comply with both Regulation 1/2005 on the protection of animals during transport and Regulation 561/2006 on the harmonization of certain social legislation relating to road transport. The latter provides for maximum driving periods and minimum resting periods for road drivers. The legislation to protect animals during transport and the legislation to protect drivers are not fully congruent. CJEU still considers that the requirements of the two pieces of legislation can be applied (CJEU case C-469/14). A driver is allowed to drive for 4.5 h before having a break of 45 min and repeat that without exceeding a total of 14 h before a 1-h break, and 29 h before a 24-h break during which animals must be unloaded, watered, and fed (Duthoit, 2017; Figure 3).

Applying EU legislation to transport that end in a third country seems difficult at present. The Netherlands decided in 2020 to no longer approve the export of live animals if the journey includes a mandatory break at a control post (i.e where animals are unloaded to rest) outside the EU, as the authorities lack information on the compliance with the Regulation (Eurogroup for Animals, 2020a). As far as we know, except for a few German landers, no other MS took the same decision.

## The Way Forward: From Simple Steps to Huge Leaps

### Increase and improve inspections

Paladino et al. (2021) recommended that on-road inspections should be increased, with traffic police officers being trained in enforcing Regulation 1/2005. Official veterinarians should support traffic police during on-road inspections. A report from the NGO Animals’ Angels (2021) added that veterinary inspections at loading should be systematic, that more inspections should be unannounced and asked for improved harmonization among MS.

Inspectors can be agents of change to lead to compliance (Overstreet and Anneberg, 2020). Empathy and support may be more effective in engaging farmers to change than cold-hearted and distant communication for strict enforcement of the regulation (Overstreet and Anneberg, 2020). Herskin et al. (2020) noted that Regulation 1/2005 is “*not rigorous*”, therefore, anyway, “*compliance does not equate to good animal welfare*”. They proposed instead that all relevant stakeholders be engaged together to find concrete solutions to improve animal welfare and compliance.

### Cover all farm animal species

Aquatic vertebrates are covered by Regulation 1/2005 but fish are not mentioned and there are no specific requirements for their transport. Some of the general provisions are “*neither appropriate nor necessarily properly implemented because they have been developed on the basis of approaches taken for terrestrial animals*” (Hedley and Huntington, 2009). This is for instance the case for feeding animals during transport, as “*feeding fish prior to or during transport quickly leads to poor welfare and death of the transported animals, mainly because of changes in water quality in transport tanks*” (Hedley and Huntington, 2009). According to the European Food and Safety Agency (EFSA, 2004), “*the duration of transport, stocking densities, and environmental conditions during the process can result in deterioration in the welfare, including the health, of the particular fish species*”.

In our opinion, the EU regulation on the protection of animals during transport should apply to every animal transported for commercial purposes including invertebrate aquatic animals and should provide species-specific requirements for all species covered.

### Set up reduced maximum journey time

Reducing animal transportation time would have positive effects on animal welfare (Frisk et al., 2018). EFSA (2011) recommends “*reducing journey time (e.g. by slaughtering animals as close as possible to the site of production)*”. The Federation of Veterinarians of Europe (FVE) (2008; 2016;2019) has long stated that “*animals should be reared as close as possible to the premises on which they are born and slaughtered as close as possible to the point of production*”. Eurogroup for Animals (2021) recommends to restrict traveling time to 8 h for adult bovines, ovine, and pigs, and to 4 h for poultry, rabbits, younger animals (except unweaned animals whose transport should be forbidden), and animals at the end of the production cycle. Eurogroup for Animals (2021) also recommends banning the transport of animals at 40% or more of pregnancy. We believe that there is an urgent need to reduce the maximum duration allowed for a journey and to avoid transporting unweaned animals or pregnant females after a certain stage of pregnancy.

### Phase out livestock exports

EFSA (2011) recommends to “*reduce the volume of transport (e.g. replacing the transport of breeding animals by using semen or embryos)*” and to reduce “*long distance transport of animals for finishing or slaughter (e.g. by the transport of carcasses and food products)*”. FVE also calls for “*replacing the transport of live animals by the transport of carcasses/ animal products*” (FVE, 2008; 2016; 2019). Most animal welfare groups like Eurogroup for Animals (2021) also call for the replacement of live terrestrial farm animal export with the export of meat, carcasses, semen, and embryos.

At the EU institutions side, the European Parliament (2019)*“[called] on the Commission to develop a strategy to ensure a shift from live animal transport to a mainly meat-and-carcass and germinal products trade*”. The Council of the EU (2019) called “*for more discussion in different forums concerning the sustainability of trade in live animals versus meat*”. In 2021, the Netherlands, Germany, and Luxembourg jointly called “*for an EU-wide ban on the long-distance transport of livestock to third countries, both by land and by sea*” (Council of the European Union, 2021).

In a nonpeer-reviewed study commissioned by several animal welfare organizations, Baltussen et al. (2017) compared the sustainability of live animal transport with the transport of meat. They based their models on available information related to costs of transport, slaughter, technical differences, CO_2_ emissions, as well as consumer preferences, animal welfare, and employment. They applied it to two case studies: hens transported from the Netherlands to Poland and lambs transported from Hungary to Italy. While the results were not conclusive for the case study on hens (the transport of live hens is sustainable economically, but is not environmental- and animal welfare-friendly), the second case proved to be much more cost-efficient from all variables with the transport of meat rather than the transport of live lambs. Besides, the transport of meat is already a common practice within the EU and to and from third countries: in 2018, the trade of meat and edible meat offal among MS amounted for about €37 billion intra-EU and for about €14 billion outside the EU, while live animal trade represented respectively €8.6 billion and less than €3 billion (Rossi, 2020).

We suggest that replacing the transport of livestock with the transport of carcasses, semen and embryos could improve significantly the welfare of livestock in the EU.

## Conclusion

Transport may result in poor welfare for an animal if it is not carried out in good conditions. Regulation 1/2005 aims to protect animals during transport. The Regulation brought some positive changes for the animals. Yet, investigations from NGOs, reports from EU institutions and MS, and scientific literature revealed the limits of the regulation to satisfactorily protect the animals, whether it be due to the content of the Regulation or to deficiencies in its enforcement. In its 2019 conclusions, the Council of the EU “*[stressed] the need to improve the welfare of animals during transport over long distances [… and encouraged] the Commission to review and update Regulation (EC) No 1/2005*” (Council of the European Union, 2019). In late 2020, the European Parliament set up a Committee of Inquiry on the Protection of Animals during Transport to investigate the issue and make proposals to improve the regulation. In the Farm to Fork strategy, the European Commission announced that it “*will revise the animal welfare legislation, including on animal transport […]*” (European Commission, 2020). In our opinion, the most impactful solution would be a shift from live animal export to the transport of meat and carcasses. This could be both economically viable and environmentally sustainable. New Zealand has already partially banned the export of livestock for slaughter (with derogations) (Animal Welfare (Export of Livestock for Slaughter) Regulations 2016, LI 2016/172) and now plans to totally ban the export of cattle, deer, sheep, and goats by sea for any purpose from 2023 (Animal Welfare Amendment Bill, 2021). Similarly, the United Kingdom intends to ban livestock export for slaughter from England and Wales (Animal Welfare (Kept Animals) Bill, 2021). We also believe that imposing maximum journey duration for each species would significantly improve animal welfare. In the meantime, we suggest that detailing specific provisions for each species and stage of life as well as increasing and improving inspections would have positive impacts on the welfare of animals transported in the EU.

## About the Author

**Nikita Bachelard** is Public affairs Coordinator at La Fondation Droit Animal, Éthique et Sciences (LFDA), a French animal welfare organisation. She holds a Master’s degree in Political Sciences and International Relations from Sciences Po Toulouse, France. Nikita first worked for the animal protection group Animal Defenders International for several months before joining LFDA’s team in 2017. As part of her position, she is in charge of the lobbying activity, including at the European Union level.
